# Pencil beam scanning proton therapy for mediastinal lymphomas in deep inspiration breath-hold: a retrospective assessment of plan robustness

**DOI:** 10.2340/1651-226X.2024.23964

**Published:** 2024-02-28

**Authors:** Filip Hörberger, Karin M. Andersson, Marika Enmark, Ingrid Kristensen, Anna Flejmer, Anneli Edvardsson

**Affiliations:** a Radiation Physics, Department of Hematology, Oncology and Radiation Physics, Skåne University Hospital, Sweden; b Department of Medical Physics, The Skandion Clinic, Uppsala, Sweden; c Department of Clinical Sciences, Oncology, Lund University, Lund, Sweden; d Department of Immunology, Genetics and Pathology, Uppsala University, Uppsala, Sweden; e Department of Oncology, Uppsala University Hospital, Uppsala, Sweden; f Medical Radiation Physics, Department of Clinical Sciences Lund, Lund University, Lund, Sweden

**Keywords:** Proton therapy, mediastinal lymphomas, deep inspiration breath-hold, Robust optimization, repeated verification CTs

## Abstract

**Purpose/background:**

The aim of this study was to evaluate pencil beam scanning (PBS) proton therapy (PT) in deep inspiration breath-hold (DIBH) for mediastinal lymphoma patients, by retrospectively evaluating plan robustness to the clinical target volume (CTV) and organs at risk (OARs) on repeated CT images acquired throughout treatment.

**Methods:**

Sixteen mediastinal lymphoma patients treated with PBS-PT in DIBH were included. Treatment plans (TPs) were robustly optimized on the CTV (7 mm/4.5%). Repeated verification CTs (vCT) were acquired during the treatment course, resulting in 52 images for the entire patient cohort. The CTV and OARs were transferred from the planning CT to the vCTs with deformable image registration and the TPs were recalculated on the vCTs. Target coverage and OAR doses at the vCTs were compared to the nominal plan. Deviation in lung volume was also calculated.

**Results:**

The TPs demonstrated high robust target coverage throughout treatment with *D*_98%,CTV_ deviations within 2% for 14 patients and above the desired requirement of 95% for 49/52 vCTs. However, two patients did not achieve a robust dose to CTV due to poor DIBH reproducibility, with *D*_98%,CTV_ at 78 and 93% respectively, and replanning was performed for one patient. Adequate OAR sparing was achieved for all patients. Total lung volume variation was below 10% for 39/52 vCTs.

**Conclusion:**

PBS PT in DIBH is generally a robust technique for treatment of mediastinal lymphomas. However, closely monitoring the DIBH-reproducibility during treatment is important to avoid underdosing CTV and achieve sufficient dose-sparing of the OARs.

## Introduction

In recent decades, treatment for mediastinal lymphomas has greatly improved, combining both chemotherapy and radiotherapy (RT) resulting in cure rates above 80–90% [1]. As many patients become long-time survivors the late toxic effects of RT, such as secondary primary cancer and cardiovascular disease, are of concern [2, 3]. To address this, the current focus of mediastinal lymphoma treatments has been to achieve high disease control while simultaneously reducing the side effects by decreasing the irradiated volume and the prescribed dose [4–6]. Utilizing protons, with their well-defined range and sharp dose-drop at the end of their tracks, a high dose conformity to the target can be obtained as well as further organs at risk (OARs) sparing compared to photon therapy [7, 8]. Because of these properties, proton therapy (PT) is expected to have a positive impact in the treatment of mediastinal lymphomas compared to photon RT [9–11].

However, pencil beam scanning (PBS) PT in the thorax region imposes major challenges as respiratory motion can severely degrade the dose distribution because of interplay effects and/or density variation altering the range of the protons [12–14]. To adhere to these challenges, robust optimization (RO) and motion-mitigation techniques, for example, Deep Inspiration Breath-Hold (DIBH), can be used. If successfully applied, DIBH has the potential to generate a geometrical situation that is almost static as well as reducing the dose to OARs (particular the heart and lungs) and normal tissue because the target volume is placed in a more favorable position throughout irradiation [5, 6, 15].

The dosimetric benefits of utilizing PT with DIBH for treatment of mediastinal lymphomas in comparison with photon radiotherapy have been demonstrated in several studies [5, 15, 16]. The aim of this study was to describe the clinical procedure of PBS-PT in DIBH for mediastinal lymphoma patients and to retrospectively evaluate plan robustness to the target and OARs by weekly CT images acquired throughout treatment. To our knowledge, this is the most comprehensive evaluation of plan robustness throughout treatment for PBS-PT in DIBH for mediastinal lymphomas reported.

## Material and methods

### Patients

Sixteen patients diagnosed with mediastinal lymphoma (8 females and 8 males, median age 27 years) who underwent PT in DIBH were enrolled in this study. This corresponds to all patients treated between February 2018 and February 2022, excluding one patient who actively declined participation in the study. Patient characteristics are presented in Table 1.

**Table 1:** Patient characteristics.

Patient	Gender [M/F]	Target location	CTV volume [cm^3^]	Total dose (Gy[RBE])	Fractionation (Gy[RBE] × fractions)
1	M	Mediastinum	130	20.00	2.00 × 10
2	F	Mediastinum + left SF	72	20.00	2.00 × 10
3	F	Mediastinum + right SF	367	29.75	1.75 × 17
4	F	Mediastinum	126	29.75	1.75 × 17
5	M	Mediastinum	169	36.00	1.80× 20
6	F	Mediastinum	158	29.75	1.75 × 17
7	M	Mediastinum + right SF	311	29.75	1.75 × 17
8	M	Mediastinum	167	29.75	1.75 × 17
9	F	Mediastinum + right SF and right neck	144	29.75	1.75 × 17
10	F	SF + right axilla	275	29.75	1.75 × 17
11	M	Mediastinum + left SF	680337150	30.60	1.80 × 11Boost: 1.80 × 6
12	F	Mediastinum	142	30.00	2.00 × 15
13	M	Mediastinum + left SF	393	30.00	2.00 × 15
14	F	Mediastinum	30318	36.00	2.00 × 15Boost: 2.00 × 3
15	M	Mediastinum	765	30.00	2.00 × 15
16	M	Mediastinum	289	40.00	2.00 × 20

SF: Supraclavicular fossa; CTV: clinical target volume.

Treatment was given at the national proton facility in Sweden, the Skandion Clinic in Uppsala; however, all preparatory work including immobilization, CT-scanning and treatment planning was performed at Skåne University Hospital [17]. The clinical plans used for treatment were retrospectively evaluated in this study.

The selection, preparation, plan optimization and treatment delivery for all patients were in accordance with national protocol [4].

### CT-imaging and deep inspiration breath hold

Planning computer tomography images (pCT) in DIBH were acquired for all patients with Siemens SOMATOM Definition AS CT scanners (Siemens Medical Solutions, Erlangen, Germany). In general, the patients were positioned headfirst supine with both arms above the head. One patient was positioned with one arm up and another patient with a thermoplastic mask and arms down. For DIBH, the vertical motion of the chest wall was tracked with the surface scanning system Sentinel™ (C-rad Positioning AB, Uppsala, Sweden). The gating point was placed on the lower part of sternum and the chest-motion during a breath-hold was limited to 3 mm in the gating window. All patients were instructed to reach a comfortable and reproducible chest-amplitude as well as stay in the gating window for about 20 s. Audio instructions on when to inhale/exhale were given to the patients and the patients could monitor their breathing with video goggles. The same approach was used during treatment. In addition to the pCT, two DIBH-CTs were acquired at the same session immediately after the pCT to evaluate inter breath-hold variations. The patients were instructed to perform training breath-holds between the pCT and the additional CT-scans in order to simulate treatment conditions [10].

The clinical workflow has previously been described in more detail by Andersson et al. [15].

### Contouring and treatment planning

The gross tumor volume (GTV) was delineated as the fluorodeoxyglucose positron emission tomography positive residual tumor growth. The clinical target volume (CTV) was defined as the original spread of the disease including the GTV and it was verified that the CTV was encompassed in the repeated DIBH-CTs. Delineated OARs included: heart, combined volume of left and right lung, esophagus and the breasts for the female patients.

Treatment plans were created in Eclipse™ treatment planning system (TPS) (Varian Medical Systems, Milpitas, CA). The plans were robustly optimized on the CTV with the optimizer algorithm Nonlinear Universal Proton Optimizer (Version 13.7.15-15.6.03) and the Proton Convolution Superposition dose calculation algorithm (Version 13.7.15-15.6.04). A constant relative biological effectiveness (RBE) value of 1.1 was assumed [18, 19]. In general, two to three anterior oblique fields (gantry angles 335°–25°) were used depending on optimal beam entrance with respect to target, OARs and to minimize density variation in the beam path. For one patient, a posterior field was necessary to achieve a robust dose to CTV and optimal OAR sparing. A range shifter (water-equivalent thickness of 3.5 cm) was generally used to increase the spot size [20] as this has been demonstrated to reduce interplay effects [21]. Spot-spacing varied between 0.3 and 0.7 cm. Single field uniform dose optimization was used for all patients, except for patient 9 where multifield optimization was used. TPs were robustly optimized with perturbations of up to 7 mm set-up displacement (corresponding to our clinical CTV to planning target volume margin for these tumors) and 4.5% range uncertainty [22]. Before plan acceptance, all TPs were robustly evaluated with 7 mm/4.5% to validate sufficient target coverage and OAR sparing. However, patient 2 was clinically evaluated with 5mm/4.5%. According to criteria used in our clinic, 98% of the CTV should receive at least 95% of the prescribed dose (*D*_98%,CTV_ ≥ 95%) for 10/12 uncertainty scenarios, with the aim to fulfill the criteria for as many scenarios as possible.

Based on national guidelines [4], the following dose volume histogram (DVH)-parameters were analyzed: *D*_98%,CTV_, *D*_Heart_, *D*_Lungs_, *D*_L/R Breast_, *D*_2%,Esophagus_ and *V*_15Gy(RBE),Heart_, *V*_5Gy(RBE),Lungs_, *V*_5Gy(RBE),L/R Breast_ and *V*_20Gy(RBE),Lungs_.

### Treatment delivery

The dedicated PBS facility uses a Proteus Plus PT system (Ion Beam Applications, Louvain-la-Neuve, Belgium), with beam energies from 60 to 226 MeV. The patients were initially positioned in free-breathing with the optical surface scanning system Catalyst^TM^ (C-rad Positioning AB, Uppsala, Sweden). After the initial patient positioning, orthogonal X-ray images were acquired in DIBH, and patient position was potentially corrected for with up to six degrees of freedom. During treatment, the Catalyst^TM^ system was used for tracking the respiratory motion and beam gating.

From the Catalyst^TM^ log-files the breath-hold amplitude (distance from the baseline to the lower end of the gating window), the number of breath-holds during each fraction (including imaging and treatment), and the session time were extracted. Session time was defined as the time from when Catalyst^TM^ was turned on, until the end of the last breath-hold. Finally, the total beam-on time was retrospectively obtained by simulating the beam-on time for every field in each TP.

### Verification CT:s

In the same way as during the pCT, three DIBH-CTs were acquired at the Skandion Clinic (Siemens SOMATOM Definition AS CT) prior to treatment to verify the patients DIBH-reproducibility. One of these CT-scans was also used to analyze the robustness of the TP. Repeated DIBH CT-scans were then acquired, before or after a treatment fraction, at least once a week during the treatment course. For the CT-scans acquired after a treatment fraction the patients were transported on trolley between the treatment room and CT-scanner. All CT-scans acquired during the treatment period including the DIBH CT-scan used for the robust analysis shortly prior to treatment will henceforth be referred to as verification CTs (vCT). Between 2 and 5 vCTs were acquired for each patient, resulting in a total of 52 vCTs for the entire patient cohort. The structure set for each patient was transferred from the pCT to the vCTs with deformable image registration (DIR), based on the Demons algorithm in Eclipse**™** TPS. A physician experienced in PT reviewed each transferred structure to determine whether they were clinically acceptable or not.

Dosimetric variations throughout treatment were determined by recalculating the original TP on the vCTs. Target coverage (*D*_98%,CTV_) and OAR doses were compared with the nominal plan values. The total lung volume variation between the pCT and vCTs was also computed.

## Results

### Treatment delivery

Treatment and breath-hold characteristics are presented in Table 2. The median breath-hold amplitude was 10.1 mm (range 8.0 – 19.5 mm).

**Table 2:** Treatment characteristics for each patient, including median number of breath-holds per fraction, total beam-on time, and median session time. The ranges are presented in brackets. The median and range for the entire patient-cohort is also presented.

Patient	Breath-holds	Beam-on time [min: s]	Session time [min]
1	8 (6–15)	1:35	22 (15–46)
2	5 (3–9)	1:52	18 (14–32)
3	10 (7–19)	2:39	25 (17–58)
4	7 (5–11)	1:22	19 (14–26)
5	7 (6–25)	1:41	17 (11–51)
6	12 (9–21)	2:37	32 (23–60)
7	11 (8–14)	3:06	17 (14–35)
8	13 (11–20)	2:49	24 (15–62)
9	18 (14–26)	5:22	27 (21–35)
10	11 (7–22)	2:31	25 (16–61)
11	20 (16–28)Boost: 19 (15–37)	6:55Boost: 4:21	32 (25–53)Boost: 33 (31–55)
12	10 (8–12)	2:04	21 (15–34)
13	10 (8–13)	2:35	26 (19–37)
14	13 (10–29)Boost: 7 (6–7)	3:18Boost: 0:56	24 (19–64)Boost: 15 (15–23)
15	24 (19–55)	5:09	37 (26–73)
16	10 (6–23)	2:15	26 (17–37)
MedianRange	115–24	2:331:22–6:55	2517–37

### Verification CT:s

**Figure 1:**
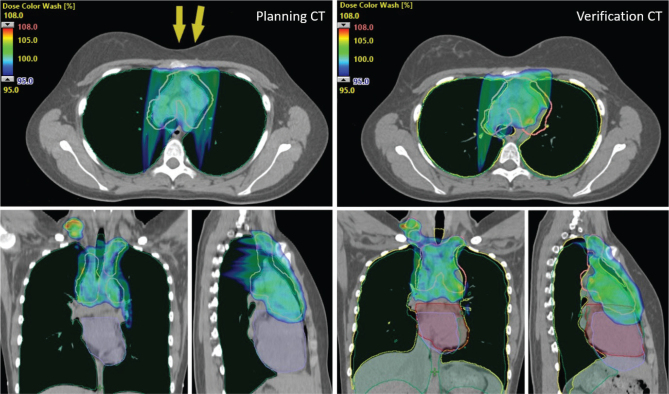
Example of dose distributions for patient 3 at the planning deep inspiration breath-hold (DIBH) CT-scan (left) and the second verification DIBH CT-scan (right). Cut-off dose is 95% of the prescribed dose (29.75 Gy[RBE]) and beam arrangement is illustrated by the yellow arrows. The clinical target volume (CTV) is marked in pink and the CTV in the verification CT has been transferred with deformable image registration. To illustrate anatomical changes, the lungs and heart have been transferred with both rigid and deformable image registration. The rigid contours have the same color as in the planning CT (green and purple for the lungs and heart, respectively), whereas the deformed lungs and heart are marked in yellow and red, respectively. Dose coverage drop to the CTV can be observed at the verification CT due to large anatomical differences, which prompted replanning.

The structures transferred by DIR were all determined to be clinically acceptable. Examples of transferred structures and dose distributions for one patient are presented in Figure 1.

**Figure 2:**
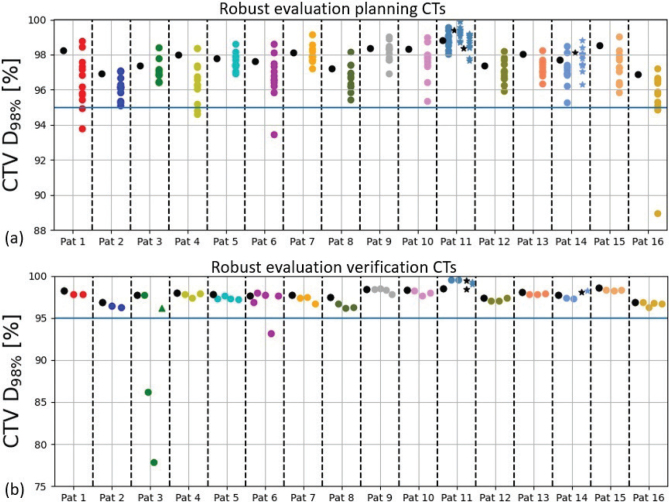
Target coverage (*D*_98%,CTV_) for the nominal plans (black points) at the planning deep inspiration breath-hold (DIBH) CT (A) with corresponding robust evaluation (7mm/4.5%, patient 2: 5mm/4.5%), where each colored point represents a different uncertainty scenario. Achieved target coverage throughout treatment (B), with the nominal plan values (black points) and the verification DIBH-CTs (colored points). The verification DIBH-CTs are presented in order of acquisition (from left to right). For both (A) and (B) the boost plans for patients 11 and 14 are represented by the stars and the blue line is the dose criteria for *D*_98%,CTV_ that is *D*_98%_ ≥ 95%. In patient 3 case replanning was performed due to low target coverage on the second verification CT and the triangle in (B) represent the new optimized treatment plan.

**Figure 3:**
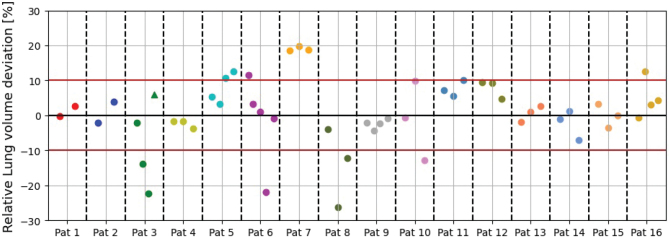
Relative total lung volume deviation between each deep inspiration breath-hold (DIBH) deformed lung-contour at the verification CTs (vCT) with the same structure from the planning DIBH-CT. The colored points represent the relative deviation at each vCT. The vCTs are presented in order of acquisition (from left to right). The triangle for patient 3 represents the lung volume deviation between the last and second vCT since replanning had been performed on the second vCT.

**Figure 4:**
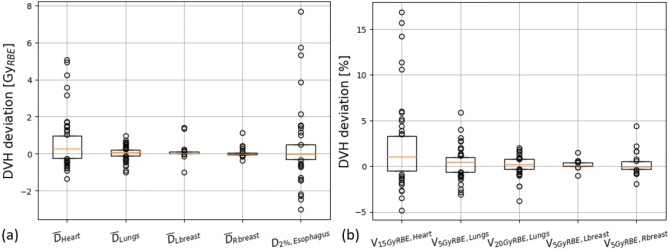
Deviation in dose volume histogram (DVH) parameters to organs at risk between the verification CTs (vCT) and the planning CT. A positive value corresponds to a higher value in the vCT plans compared with the nominal plan values. All boxplots consist of 52 datapoints, that is all vCTs, except for the breasts, which consists of 24 datapoints (only female patients). The lower and upper 25% of the dataset is presented as points to visualize the spread of the larger deviations.

For the nominal TPs, the CTV robustness was high with *D*_98%,CTV_ ≥ 95% for at least 10 out of 12 perturbations for all patients (Figure 2A). Throughout treatment plan robustness to the CTV was generally high for all recalculated plans. The *D*_98%,CTV_ deviations were within 2% for 14 patients and above the desired requirement of *D*_98%,CTV_ ≥ 95% at 49 out of 52 vCTs (Figure 2B). Only patients 3 and 6 had *D*_98%,CTV_ < 95% for two and one vCT, respectively. At these vCTs, a large lung volume deviation by up to 22% was observed (Figure 3). For patient 3, replanning was deemed necessary. Deviations in OAR DVH-parameters were small for most patients throughout the treatment course (Figure 4). The nominal OAR DVH-parameters are presented in supplementary Table 1.

The relative lung volume deviations between the vCTs and the pCT are presented in Figure 3. For the majority of the vCTs, the deviation was within ±10%; however, some patients demonstrated larger deviations of up to 28%.

## Discussion

Pencil beam scanning PT plans robustly evaluated with a 7 mm setup error and 4.5% range-uncertainty were found to deliver a robust dose to CTV throughout treatment, with *D*_98%,CTV_ ≥ 95% and deviations within 2% for the majority of the patients. Adequate OAR sparing was achieved both for the nominal plans and for the plans recalculated on the weekly vCTs, meeting our national guidelines [4] as well as the guidelines presented by the International Lymphoma Radiation Oncology group [7]. This indicates that PBS-PT in DIBH is a suitable treatment technique for mediastinal lymphomas.

In accordance with clinical practice, if a degraded dose distribution is observed in a vCT a follow-up vCT is promptly acquired to determine whether the degradation is temporary or persistent and to assess the need for replanning. As the second vCT for patient 3 revealed subpar target coverage, a subsequently vCT was promptly obtained and since this one also exhibited inferior target coverage replanning was deemed necessary. The new TP was optimized based on the second vCT, resulting in satisfactory target coverage in the final vCT (Figure 2B). Had replanning not been performed the *D*_98%,CTV_ would have been 92.0% for the final vCT. Contrary, a *D*_98%,CTV_ of 96.4% was achieved with the new TP. Regarding patient 6, inadequate target coverage was also observed for one of the vCT (fourth). However, as the subsequent vCT exhibited desirable target coverage and OAR-sparing replanning was not considered necessary.

At the vCTs for patients 3 and 6 where *D*_98%,CTV_ was below 95%, a large deviation in lung volume by up to 20% was also observed. However, a few other patients still attained high target coverage despite large lung volume deviation at some vCTs (Figures 3 and 4). One explanation could be that target coverage to some CTV locations within the mediastinal region are more susceptible to lung volume variation than others, and this should be further investigated.

The largest OAR dose deviations were seen for the heart (*D* and *V*_15Gy(RBE)_) and esophagus (*D*_2%_). At five vCTs (three patients) the increase in *D*_Heart_ or *V*_15Gy(RBE),Heart_ was larger than 3 Gy(RBE) and 10% respectively. For all these vCTs a smaller lung volume was observed compared to the pCT, decreasing the separation between the target and heart thus increasing the heart dose, as shown in previous studies [5, 16]. Larger *D*_2%_ deviations to the esophagus (3.8 Gy[RBE]) were only observed for patient 16.

The findings in this study highlight the necessity of closely evaluating the DIBH reproducibility throughout the treatment course to avoid underdosing the CTV and/or overdosing OARs. Daily 3D verification imaging is the recommendation by The Particle Therapy Co-Operative Group regarding PT in DIBH for mediastinal lymphomas [16] in order to confirm the DIBH reproducibility. However, they highlight the limitation of current image guided radiotherapy, that is cone-beam CT (CBCT), with respect to acquisition time relative to the breath-hold duration. We agree that, ideally, the interfractional breath-hold variations should be monitored with daily 3D verification images or, when not available, at minimum with weekly CTs (and when considered necessary), which is recommended by the American Association of Physicists in Medicine Task Group Report 290 [23]. At our institution it is practically difficult to acquire CBCT in DIBH and as such weekly CTs is the most applicable solution.

Studies including mediastinal lymphoma patients treated with PBS-PT in DIBH or free breathing (FB) are still limited [8, 11, 15, 24–27]. Furthermore, most previous studies have focused on dosimetric comparisons between proton and photon RT or radiation toxicity. König et al. [11] reported a significant reduction in dose to the heart, lung, breasts, esophagus, and spinal cord with PT compared to IMRT in a cohort of 20 patients treated in FB. Ntentas et al. [24] also demonstrated similar result with a significant reduction in mean dose to the same OARs (except the heart) with PBS-PT compared to IMRT or 3D conventional radiotherapy for 21 patients treated in DIBH. Furthermore, Hoppe et al. [26] reported a 3-year relapse-free survival rate of 92% and no acute and/or late grade 3 toxicity for 138 patients with Hodgkin lymphoma treated with PT, whereby 17 patients treated with the PBS-technique. Tseng et al. [27] reported a low rate (13%) of symptomatic radiation pneumonitis and no grade 3 or higher radiation pneumonitis for a cohort of 85 patients treated with PT, whereby 24 patients treated with the PBS-technique.

Most previous studies, however, do not report about plan robustness throughout treatment which is one of the major concerns with PT as anatomical changes or breath-hold variability can severely degrade the resulting dose-distribution. In the study by Righetto et al. [25], which included 14 patients treated with PBS-PT in DIBH, one repeated CT was acquired about halfway through treatment for all patients to assess the dosimetric impact from interfractional variations. In accordance with our study, they concluded that the variations were within the margins used (6mm/3.5%) and produced only minimal dosimetric impact on target coverage and the OARs throughout the treatment. Similarly, Andersson et al. [15] reported minor dose deviations in both *D*_98%,CTV_ and OARs (below 0.6 and 2%, respectively) for two patients treated with PBS-PT in DIBH. As such, the strength of this study is the extensive evaluation of plan robustness with a large number of vCTs acquired throughout treatment. Additionally, this study evaluates both anatomical and dosimetric differences due to DIBH-variability and DIR was used which eliminates inter-physician delineation differences.

Before plan acceptance, all TPs were determined to be robust in both target coverage and adequate OAR-sparing against a 7 mm setup error and 4.5% range-uncertainty. For most patients, the plans were robust throughout treatment, however, insufficient target coverage was observed for two patients due to large anatomical differences resulting from DIBH-variability. It can be assumed that for these patients the target dose-coverage loss would have been observed regardless of the perturbations used in the robust optimization (RO). Lower perturbations have been reported in a previous study (6mm/3.5% by Righetto et al. [25]) demonstrating sufficient plan robustness. Although the evidence for plan robustness throughout treatment with PBS-PT in DIBH for treatment of mediastinal lymphoma is still limited, lower perturbations in the RO, potentially in combination with daily 3D verification imaging, should be investigated in the future to achieve further OAR dose sparing and a further reduction of the total integral dose.

In this study, DIBH was used to increase the distance between the target and OARs, as well as to minimize respiratory motion and prevent unwanted interplay, which is important as the overall treatment quality might be compromised from these effects [7, 28]. Meijers et al. [29] assessed that with PBS-PT for lymphoma patients, the loss of dose homogeneity during a single fraction was ‘smeared out’ when averaged over a full fractionated treatment, resulting in minimal dosimetric impact from interplay effects. Additionally, Zeng et al. concluded in two studies [21, 30] that for mediastinal lymphomas, the impact of interplay effects on PBS plan robustness can be minimized if volumetric repainting and/or large spot size are employed. The findings from those studies were derived from 4D-CT scans acquired in FB, and one would expect treatment in DIBH to further prevent interplay effects since motion is restricted within the gating window (3 mm in this study) [31]. Furthermore, a range-shifter was used for most treatment fields in this study (also for non-superficial targets) to increase the spot size [20]. Volumetric repainting was not used; instead, several fields with a small angular displacement were used to increase the robustness. The use of repainting could be a disadvantage for treatment in DIBH since it prolongs the treatment delivery time and hence the number of breath-holds required.

Patients must undergo a series of breath-holds during each fraction and in this study a variation in the number of breath-holds and session time was observed for all patients with some treatment fractions requiring a larger number of breath-holds (Table 2). If patients become fatigued during a treatment fraction the DIBH-reproducibility may be compromised, potentially resulting in a systematic target dose-coverage loss and inadequate OAR sparing. Furthermore, all vCTs in this study were acquired during a single breath-hold, before or after a treatment fraction, and the assumption was that this breath-hold was representative of the breath-holds performed during treatment. The reason to acquire some vCTs after a treatment fraction was to establish the DIBH-reproducibility at the end of a breath-hold series. Intrafractional- and interfractional breath-hold variations were evaluated to some degree by acquiring a series of DIBH-CTs on two separate occasions (at the time of pCT acquisition as well as shortly prior to the first treatment fraction). Despite this, there may still be intrafractional- and interfractional breath-hold variations which were not accounted for in this study. Future research investigating the dosimetric effects of intrafractional breath-hold variations during treatment delivery, in particular for treatment fractions requiring a large number of breath-holds where these variations would likely be the largest and most detrimental, are needed. To our knowledge, such studies are currently not published. DIBH-reproducibility at the end of a breath-hold series is a concern [23] and therefore consideration with respect to the number of fields, monitor units, etc. should be made in order to keep the number of required DIBHs to a minimum.

Several studies and guidelines have recommended the use of 4D robust optimization (4D-RO) to mitigate DIBH-variability or other inter/intra fractional variations [16, 23, 32]. With 4D-RO, the repeated DIBH CT-scans are incorporated in the optimization process itself, but as of today, it has not yet been completely implemented in commercial TPSs [23]. The large DIBH-variability seen during the treatment course in this study for two patients was not representative of the repeated DIBH-scans acquired at the pCT session and as such employing 4D-RO would not have been sufficient in generating robust target coverage in these cases.

A constant RBE value of 1.1 was assumed as per current clinical practice in PT [19, 33]; however, in reality the RBE increases at the end of the proton range [34]. For treatment of mediastinal lymphomas, anterior fields could range into the OARs situated behind the target and potentially increase the biological effective dose to these organs. In a recent study by Rechner et al. [35], TPs with a variable RBE-weighted (vRBE) dose were investigated for mediastinal Hodgkin lymphomas using similar field arrangement as in this study. The near-maximum doses to the heart and cardiac substructures increased on the order of 2–3 Gy with the vRBE compared with a fixed RBE of 1.1. They recommended caution when near-maximum doses are near tolerance levels, which was not the case in this study.

## Conclusion

This evaluation of repeated vCTs showed that PBS PT in DIBH is a feasible and robust technique for treatment of mediastinal lymphomas. However, it is important to closely evaluate the DIBH reproducibility during the treatment course to avoid underdosing CTV and to achieve sufficient dose sparing of the OARs.

## Supplementary Material

Pencil beam scanning proton therapy for mediastinal lymphomas in deep inspiration breath-hold: a retrospective assessment of plan robustness

## Data Availability

The authors confirm that the findings of this study are available from the data presented in the article.
