# Supplementary material for: Pencil beam scanning proton therapy for mediastinal lymphomas in deep inspiration breath-hold: a retrospective assessment of plan robustness
Source: Acta Oncol. 2024 Feb 28;10:23964. doi: 10.2340/1651-226X.2024.23964 (PMC11332452; doi:10.2340/1651-226X.2024.23964)
Supplement: Pencil beam scanning proton therapy for mediastinal lymphomas in deep inspiration breath-hold: a retrospective assessment of plan robustness [file AO-63-23964-s1.pdf]

Supplementary material has been published as submitted. It has not been copyedited or typeset by Acta Oncologica.

Supplementary table 1: Nominal organs at risk dose-volume parameters.

Patient	Heart $\overline{D}$ [Gy(RBE)]	Heart $V_{15Gy(RBE)}$ [%]	Lungs $\overline{D}$ [Gy(RBE)]	Lungs $V_{5Gy(RBE)}$ [%]	Lungs $V_{20Gy(RBE)}$ [%]	Esophagus $D_{2\%}$ [Gy(RBE)]	L Breast $\overline{D}$ [Gy(RBE)]	L Breast $V_{5Gy(RBE)}$ [%]	R Breast $\overline{D}$ [Gy(RBE)]	R Breast $V_{5Gy(RBE)}$ [%]
1	0.90	1.40	0.7	4.5	0.00	15.74	-	-	-	-
2	0.36	0.10	1.4	8.90	0.30	16.84	0.00	0.00	0.00	0.00
3	4.43	12.90	5.3	24.10	14.10	29.45	6.47	32.30	1.09	6.90
4	3.63	10.60	3.4	17.90	7.00	25.83	1.10	7.80	2.32	16.70
5	4.96	13.80	3.2	14.40	6.80	10.43	-	-	-	-
6	4.75	15.20	3.06	15.10	7.40	28.32	0.31	2.20	1.19	7.80
7	3.11	9.00	3.00	15.90	7.80	29.93	-	-	-	-
8	6.22	18.90	3.36	17.20	7.10	28.32	-	-	-	-
9	1.00	1.10	3.15	14.00	8.50	30.35	0.03	0.10	2.71	13.10
10	0.49	0.50	3.45	17.10	8.30	1.03	0.05	0.20	9.75	43.80
11	10.42	40.60	5.93	37.90	1.40	20.11	-	-	-	-
12	4.19	11.90	2.95	14.90	6.60	29.55	1.59	8.70	0.13	0.80
13	7.51	23.20	4.77	26.00	9.20	29.07	-	-	-	-
14	12.48	40.60	6.48	35.30	13.00	27.48	-	-	-	-
15	11.59	37.10	6.75	43.30	10.70	29.49	-	-	-	-
16	5.03	13.00	3.75	16.30	7.60	25.51	-	-	-	-
